# ROS-dependent HIF1α activation under forced lipid catabolism entails glycolysis and mitophagy as mediators of higher proliferation rate in cervical cancer cells

**DOI:** 10.1186/s13046-021-01887-w

**Published:** 2021-03-11

**Authors:** Serena Castelli, Fabio Ciccarone, Daniela Tavian, Maria Rosa Ciriolo

**Affiliations:** 1 Department of Biology, University of Rome “Tor Vergata”, Via della Ricerca Scientifica 1, 00133 Rome, Italy; 2 IRCCS San Raffaele Pisana, Department of Human Sciences and Promotion of the Quality of Life, San Raffaele Roma Open University, Rome, Italy; 3 Laboratory of Cellular Biochemistry and Molecular Biology, CRIBENS, Catholic University of the Sacred Heart, pz Buonarroti 30, 20145 Milan, Italy; 4 Psychology Department, Catholic University of the Sacred Heart, Largo Gemelli 1, 20123 Milan, Italy; 5 IRCCS San Raffaele Pisana, Via della Pisana 235, 00163 Rome, Italy

**Keywords:** ATGL, HIF1α, Pseudo-hypoxia, Lipid catabolism, Mitophagy, ROS

## Abstract

**Background:**

In the last decades, the concept of metabolic rewiring as a cancer hallmark has been expanded beyond the “Warburg effect” and the importance of other metabolic routes, including lipid metabolism, has emerged. In cancer, lipids are not only a source of energy but are also required for the formation of membranes building blocks, signaling and post-translational modification of proteins. Since lipid metabolism contributes to the malignancy of cancer cells, it is an attractive target for therapeutic strategies.

**Methods:**

Over-expression of the adipose triglyceride lipase (ATGL) was used to boost lipid catabolism in cervical cancer cells. The cervical cancer cell line HeLa was employed as the primary experimental model for all subsequent studies. The lipolytic activity of ATGL was mimicked by caproate, a short-chain fatty acid that is efficiently oxidized in mitochondria.

**Results:**

Here, we provide evidence of the association between boosted lipid catabolism and the increased proliferation and migration capability of cervical cancer cells. These pro-tumoral effects were ascribed to the reactive oxygen species (ROS)-mediated induction of hypoxia-inducible factor-1α (HIF1α) triggered by the increased mitochondrial fatty acids (FAs) oxidation. HIF1α activation increases glycolytic flux and lactate production, promoting cell proliferation. At the same time, HIF1α increases protein and mRNA levels of its known target BCL2 and adenovirus E1B 19-kDa-interacting protein 3 (BNIP3), which in turn activates mitophagy as a pro-survival process, as demonstrated by the induction of apoptosis upon inhibition of mitophagy. These effects were mimicked by the short-chain fatty acid caproate, confirming that forcing lipid catabolism results in HIF1α induction.

**Conclusions:**

Boosting lipid catabolism by ATGL over-expression has a pro-tumor role in cervical cancer cells, dependent on ROS production and HIF1α induction. Together with the bioinformatics evidence of the correlation of ATGL activity with the aggressiveness of cervical cancer cells, our data suggest that ATGL could be a promising prognostic marker for cervical cancer and highlight the need of further investigations on the role of this lipase in cancer cells. This evidence could be exploited to develop new personalized therapy, based on the functionality of the antioxidant equipment of cancer cells, considering that ROS content could affect ATGL role.

**Supplementary Information:**

The online version contains supplementary material available at 10.1186/s13046-021-01887-w.

## Background

Metabolic plasticity enables cancer cells to switch between glycolysis and oxidative phosphorylation (OXPHOS) during tumorigenesis and metastasis in response to intracellular signaling pathways and environmental cues. However, it is still largely unknown how cancer cells orchestrate gene regulation to balance their glycolysis and OXPHOS activities. The switch from oxidative to glycolytic metabolism in the presence of oxygen -known as the “Warburg effect”- is a cancer cell strategy to survive by exploiting a rapid glucose-derived source not only for energy but also for anabolism [1]. Beyond glucose metabolism, the metabolic rearrangement of cancer also involves lipids: the balance between endogenous (synthesis) and exogenous (uptake) fatty acids (FAs) is required in cancer for membranes formation, signaling, post-translational modification of proteins as well as for energy supply through OXPHOS [2–4]. Cells store FAs inside lipid droplets (LDs), complex organelles characterized by a phospholipid layer surrounding a core of triacylglycerols [5]. Despite being long considered inert energy reservoirs, LD dynamism has been recently disclosed responsible for activating several signaling pathways by regulating intracellular FAs [6, 7]. Dysregulations of many enzymes that modulate FAs availability (i.e., lipogenesis, lipolysis) have been associated with tumorigenesis and tumor progression [8]. In particular, all lipases involved in LDs degradation have been associated with cancer and/or related comorbidities (e.g., cachexia) [9, 10]. Adipose triglyceride lipase (ATGL), the first and rate-limiting enzyme of the lipolytic cascade [5], has been found altered in several human cancers [2, 3]. ATGL activity, mediated by the Ser47-Asp166 dyad [5, 11], is responsible for the hydrolysis of LDs-contained triacylglycerols in diacylglycerols and free FAs, in turn promoting the activity of the downstream lipases, hormone-sensitive lipase (HSL) and monoacylglycerol lipase (MAGL) [2, 5]. FAs derived from lipase activity feed mitochondrial metabolism, boosting the OXPHOS and consequently the production of reactive oxygen species (ROS). Cancer cells exploit the connection between cellular metabolism and redox homeostasis for their proliferative advantage. In tumor, the levels of ROS are increased, and an enhancement of antioxidant systems occurs to prevent oxidative damage. Nevertheless, several lines of evidence demonstrate that ROS signaling also contributes to a switch from oxidative to glycolytic metabolism for disabling mitochondria as a harmful source of excess ROS [12, 13]. Many redox-responsive transcription factors orchestrate ROS-dependent regulation of cell cycle, metabolism, differentiation and death in cancer [14–16]. Among these, hypoxia-inducible factor-1α (HIF1α) represents the key regulator of glycolysis: it is transcribed upon intracellular ROS increase, containing functional antioxidant response elements in its enhancer [17, 18]. HIF1α activity is pointed to restore oxygen homeostasis; among the mechanisms that it employs to promote adaptations to hypoxia, HIF1α forces the metabolic switch from OXPHOS toward glycolysis [19–21]. Considering lipids as the unique energetic substrates that necessarily require mitochondria for their utilization and lipolysis as the way for mobilizing LDs-contained FAs, we over-expressed ATGL to monitor the adaptation of cervical cancer cell lines under forced lipid catabolism and identified a pro-tumor role of ATGL activity. ATGL-mediated lipid catabolism increased ROS production, which in turn promoted HIF1α activation. HIF1α increased the proliferation rate and “Warburg effect”, as well as it triggered BCL2 and adenovirus E1B 19-kDa-interacting protein 3 (BNIP3)-mediated mitophagy as a survival mechanism.

## Materials and methods

### Materials

2-deoxyglucose (2-DG) (D6134), Albumin (A3782), Chloroquine (C6628), CoCl2 (60818), Cycloheximide (CHX) (C7698), Dimethyl sulfoxide (DMSO, 154938), EDTA (E6758), EGTA (E4378), KH_2_PO_4_ (A0261677), MG-132 (474790), MgCl_2_ (A748033 012), MOPS (M3183), N-acetylcysteine (NAC, A7250), Nicotinamide adenine dinucleotide hydrate (NAD+) (N7004), TRI Reagent (T9424), Oil Red-O (ORO) (O0625), Formalin solution neutral buffered 10% (HT5012), Sodium caproate (C4026)**,** Sodium deoxycholate (D6750), Sodium orthovanadate (S6508), Sodium pyrophosphate tetrabasic decahydrate (30411), Sucrose (S0389), Trichloroacetic acid (TCA) (T6399) and Triton X-100 (T9284) were from Sigma-Aldrich. Trypan Blue 0.4% solution (17-942E) was from Lonza. Goat anti-mouse (172–1011) and anti-rabbit (172–1019) IgG (H + L)-horseradish peroxidase conjugated were from Bio-Rad Laboratories. Alexa Fluor™ 568 donkey anti-mouse IgG (H + L) (A10037), Alexa Fluor® 594 goat anti-rabbit IgG (H + L) (A11012), Hoechst 33342 (H1399), MitoTracker™ Green FM (MTG) (M7514) and MitoTracker™ Red FM (MTG) (M7512) were from Thermo Fisher Scientific. Polyethylenimine (PEI) (23966) were from Polysciences. HIF1A inhibitor, YC-1 (ALX-420-025) was from Enzo Life Sciences. L-lactate dehydrogenase (LDH) was from Roche Applied Science. DTT (281) and protease inhibitors cocktail from AMRESCO. TRIS-base (1610716) and Sodium Dodecyl Sulfate (SDS) (161–0300) from BioRad. Dihydroethidium (DHE) (D1168) is from Molecular probes.

### Cell lines, transfections and treatments

HeLa and Me-180 cell lines were purchased from American Type Culture Collection (ATCC). Cells were grown in Dulbecco’s modified Eagle’s medium (DMEM) 1 g/L glucose (EuroClone) supplemented with 10% fetal bovine serum (EuroClone), 10 U/ml penicillin/streptomycin (EuroClone) and 2 mM L-glutamine (EuroClone). Cells were authenticated and characterized by the supplier. Mycoplasma test was routinely carried out according to protocols from our laboratory. Cells were cultured at 37 °C in an atmosphere of 5% CO_2_ in air and plated at a 1 × 10^5^ cells/mL density for all the experiments. After 24 h plating, cells were transiently transfected with pcDNA™4/HisMaxC and pcDNA™4/HisMaxC-ATGL and pcDNA™4/HisMaxC-ATGL (Ser47Ala), for the 48 h, through the Polyethylenimine (PEI) reagent, according to manufacturer’s instructions. pcDNA™4/HisMaxC and pcDNA™4/HisMaxC-ATGL plasmids were kindly provided by Prof. Rudolf Zechner, Institute of Molecular Biosciences, Karl-Franzens-Universität Graz, Graz (Austria); pcDNA™4/HisMaxC-ATGL (Ser47Ala) has been obtained using the Phusion Site-Directed Mutagenesis Kit (Thermo Scientific). Ser47Ala mutation was introduced in ATGL cDNA using the following primers: forward 5′-CACATCTACGGCGCCGCGGCCGGGGCGCTCACGG-3′, reverse 5′-CCGTGAGCGCCCCGGCCGCGGCGCCGTAGATGTG-3′. The final construct was verified by DNA sequencing. The transfection efficiency was determined by transfecting cells with pATGL-EGFP. The percentage of transfected cells was assessed > 85%.

Cells were treated with 10 mM 2-deoxyglucose (2DG) (Sigma-Aldrich, cat. Number D6134) for 24 h, 2.5 mM and 5 mM caproate (Sigma-Aldrich cat. Number C4026) for 24 h, 30 μM chloroquine (CQ) (Sigma-Aldrich, cat number C6628) for 2 h, 10 μg/mL cycloheximide (CHX) (Sigma-Aldrich, cat. Number C7698) for 8 h, 150 μM CoCl2 (Sigma-Aldrich, cat. Number 60818) for 6 h, 50 μM dihydroethidium (DHE) (Molecular probes, cat. Number D1168) for 30 min, 2 μM MG132 (Sigma-Aldrich, cat. Number 474790) for 8 h, 200 μM MitoTracker™ Green FM (MTG) (M7514) for 30 min, 200 μM MitoTracker™ Red FM (MTG) (M7512) for 30 min, 5 mM N-acetylcysteine (NAC) (Sigma-Aldrich, cat. Number A7250) for 24 h, 100 μM YC-1 (Enzo Life Sciences, cat. Number ALX-420-025) for 24 h.

### Western blot analysis

At the end of experiment, cells were resuspended in lysis buffer (50 mM Tris-HCl, pH 7.4, 150 mM NaCl, 1 mM EDTA, 1% Triton X-100, 0.5% sodium deoxycholate, 0.1% SDS, 10 mM NaF, 5 mM Sodium Pyrophosphate, 2 mM Sodium Orthovanadate) supplemented with protease inhibitor cocktail (AMRESCO) to obtain protein lysates. The following sonication of samples was performed through the instrument of BRANSON ULTRASONICS CORPORATION. Protein concentration was determined by the method of Lowry et al. [22] before performing electrophoresis by SDS-PAGE and blotting onto a nitrocellulose membrane (Bio-Rad).

The following primary antibodies were used: Active β-catenin (Cell signaling, cat. Number 8814, diluted 1:1000), ATGL (Cell Signaling Technology®, cat. Number 2138S, diluted 1:1000), BNIP3 (Sigma-Aldrich, cat. Number B7931, diluted 1:1000), cleaved caspase-3 (Cell signaling, cat. Number 9664S, diluted 1:1000), caspase-3 (Sigma-Aldrich, cat. Number C8487, diluted 1:1000), cyclin D1 (Santa Cruz Biotechnology, cat. Number sc-6281, diluted 1:1000), DRP1 (Cell signaling, cat. Number 14647S, diluted 1:1000), DPR1-p S616 (Cell signaling, cat. Number 3455S, diluted 1:1000), GAPDH (Santa Cruz Biotechnology, cat. Number sc-47724, diluted 1:1000); GLUT-1 (Santa Cruz Biotechnology, cat. Number sc-7903, diluted 1:1000), HIF1α (Santa Cruz Biotechnology, cat number sc-10790, diluted 1:1000), HK-2 (Abnova, cat. Number H00003099-M01, diluted 1:1000), Lamin-B1 (Santa Cruz Biotechnology, cat. Number sc-377000, diluted 1:1000), LC3 (Sigma-Aldrich, cat. Number L7543, diluted 1:1000), LDH-A (Santa Cruz Biotechnology, cat. Number sc-33781, diluted 1:1000), N-cadherin (Sigma Aldrich, cat. Number C-3678, diluted 1:1000), PDHE1α-p S300 (Sigma-Aldrich, cat. Number AP1064, diluted 1:1000), TFAM (Santa Cruz Biotechnology cat. Number 166965, diluted 1:1000), TOM20 (Santa Cruz Biotechnology, cat. Number sc-11021, diluted 1:1000), Ubiquitin (Sigma-Aldrich, cat. Number MAB1510, diluted 1:1000), β-Actin (Cell Signaling Technology®, cat. Number #4970S, diluted 1:1000). Through the incubation with specific secondary antibodies (Bio-Rad), the signaling was identified using a Fluorchem Imaging System (Alpha Innotech), after incubation with LiteAblot® TURBO (EuroClone). Fluorchem Imaging System (Alpha Innotech) was also used to perform densitometry analyses.

### Nuclear fraction isolation

Nuclear extraction was performed by incubation of cells for 20 min on ice with nuclei isolation buffer (10 mM Tris-HCl at pH 7.8, 4 mM MgCl_2_, 1 mM EDTA, 0.5 mM DTT, 1% Triton X-100, 0.25 M Sucrose, 10 mM NaF, 5 mM Sodium Pyrophosphate, 2 mM Sodium Orthovanadate); after centrifugation to isolate nuclei, they were washed with isolation buffer without Triton X-100 and centrifuged two times. Each buffer was supplemented with protease inhibitor cocktail (AMRESCO).

Total cell extracts, cytosolic and nuclear fractions were analyzed by western blot for purity determination. Antibodies against LDH-A and Lamin B1 were used as purity control of cytosolic and nuclear fraction, respectively.

### Mitochondrial fraction isolation

Following detachment and washing of the cells, mitochondrial purification was obtained by resuspending in mitochondria isolation buffer (MIB) formed by 1 mM EGTA, pH 7.4, 5 mM MOPS, 5 mM KH_2_PO_4_, 0.1% BSA and 0.3 M sucrose, 10 mM NaF, 5 mM Sodium Pyrophosphate, 2 mM Sodium Orthovanadate. Cells were disrupted mechanically with a tight pestle with 30 strokes on ice. The homogenate was centrifuged at 2500×g for 5 min at 4 °C, and the supernatant containing mitochondria was collected while the pellet was processed as before three times to enrich mitochondrial fraction. Supernatants were then centrifuged at 15,000×g for 10 min at 4 °C. The supernatant was collected as cytoplasm faction, the pellet was washed three times with MIB and then centrifuged at 15,000×g for 10 min at 4 °C.

### Quantitative real-time PCR (RT-qPCR)

RNA extraction was performed by using TRItidy G (PanReact AppliChem, cat. Number T9424) according to the manufacturer’s instructions. Synthesis of cDNA was obtained from 1 μg of total RNA by using PrimeScript™ RT Reagent Kit (Perfect Real Time) (Takara), and RT-qPCR reaction was performed by using the SYBR®Premix Ex Taq (Tli RNase H Plus) (Takara) on QuantStudio™ 3 real-time PCR System (Thermo Fisher Scientific).

RT-qPCR was performed in triplicates by using designed and tested primer with primer-BLAST (NCBI). Primers used were obtained from Sigma-Aldrich. Human primer sequences are listed used are as follow: *BNIP3* forward: 5′-TCTGGACGGAGTAGCTCCAA-3′, reverse: 5′-CTTCCTCAGACTGTGAGCTGT-3′; *Cpt1a* forward: 5′-GACTCTGGAAACGGCCAACT-3′, reverse: 5′-ATCTTGCCGTGCTCAGTGAA-3′; *E-CAD* forward: 5′-CGTCCTGGGCAGAGTGAAT-3′, reverse: 5′-TCATTATGTGTTCTCGTGCAG-3′; *Fibronectin* forward: 5′-CGACACATTCCACAAGCGTC-3′, reverse: 5′-CATTGGTCGACGGGATCACA-3′; *HIF1α*, forward: 5′-ATTTTGGCAGCAACGACACAG-3′, reverse: 5′-TTTTTCGTTGGGTGAGGGGAG-3′; *N-CAD* forward: 5′-AGGCTTCTGGTGAAATCGCA-3′, reverse: 5′-GCAGTTGCTAAACTTCACATTG-3′; *SLC2A1/GLUT-1,* forward: 5′-TTCACTGTCGTGTCGCTGTT-3′, reverse: 5′-TGAGTATGGCACAACCCGC-3′; *SLC16A1/MCT-1*, forward: 5′-TGCGTGGGTACTGGAACAAG-3′, reverse: 5′-TGCAGGTCAAATCCAAATATCGT-3′; *SLC16A3/MCT-4*, forward: 5′-CTTCGTTTTTGTGCAGGTCCC-3′, reverse: 5′-ACGAAAGCCCCAAGAATGGA-3′; *PNPLA2*, forward: 5′-TCGTGTTTCAGACGGAGAGAA-3′, reverse: 5′-CAGACATTGGCCTGGATGAG-3′; *VEGF* forward: 5′-AGGCCAGCACATAGGAGAGA-3′, reverse: 5′-ACGCGAGTCTGTGTTTTTGC-3′; *ACTB*, forward: 5′-GGCCGAGGACTTTGATTGCA-3′, reverse: 5′-GGGACTTCCTGTAACAACGCA-3′. The relative mRNA levels were determined by using the 2^−ΔΔCt^ method. The fold changes were relative to a control after normalization to the internal standard *ACTB. PNPLA2* was used as an over-expression control.

### Cell proliferation assays

Cell proliferation was evaluated by Trypan blue exclusion test procedure, by bromodeoxyuridine (BrdU) incorporation assay and MTT colorimetric assay using the Cell counting Kit-8 (CCK-8, Sigma-Aldrich cat. Number 96992) according to the manufacturer’s protocol.

For BrdU incorporation assay, cells were incubated with 10 μM BrdU for 24 h and then fixed for 30 min in ethanol:acetic acid:di-deionized water solution (18:1:1). DNA was denaturized by incubation on ice for 10 min with 1 N HCl and then 10 min with 2 N HCl. Thanks to the treatment with PBS/0.4% Triton X-100 solution for 10 min the cells were permeabilized. The blocking was done with PBS/3%BSA for 1 h. The incubation with an anti-BrdU antibody (Santa Cruz Biotechnology cat. Number sc-32323) was performed overnight, followed by 1 h of incubation with an AlexaFluor™568 donkey anti-mouse IgG (H + L) secondary antibody; nuclei were stained with 1 μg/mL of Hoechst 33342 for 10 min. Images of cells were obtained with a Delta Vision Restoration Microscopy System (Applied Precision, Issaquah, WA) equipped with an Olympus IX70 fluorescence microscope (Olympus Italia, Segrate, Milano, Italy).

### Wound healing assay

HeLa cells were seeded into 24-well plates, at strict confluence, and incubated for 24 h period in complete medium to form a confluent monolayer. The culture well surface was scratched with 200 μL pipette tip to create a “wound”. The serum-starvation was applied to block cell proliferation and wound healing was followed for 16 h. Images were captured with a Delta Vision Restoration Microscopy System (Applied Precision, Issaquah, WA).

### Fluorescence microscopy analyses

Cell medium was removed and cells rinsed with PBS and fixed with formalin solution neutral buffered 10% (4% paraformaldehyde containing) for 1 h, blocked with PBS/10% fetal bovine serum solution for 1 h and incubated overnight with an anti-HIF1α (Sigma Aldrich, cat. Number sc-10790) antibody. The secondary antibody was Alexa Fluor® 594 goat anti-rabbit IgG (H + L) (A11012) that is incubated for 1 h at room temperature; nuclei were stained with 1 μg/ml of Hoechst 33342 for 10 min. Delta Vision Restoration Microscopy System (Applied Precision, Issaquah, WA) equipped with an Olympus IX70 fluorescence microscope (Olympus Italia, Segrate, Milano, Italy) was used to acquire fluorescent images of cells.

### Oil Red-O staining

Oil Red-O staining was performed to identify the content of lipid droplets inside the cells as previously described [23]. Briefly, culture medium was removed, and cells were washed in PBS. Cells were fixed with formalin solution neutral buffered 10% (4% paraformaldehyde containing) for 10 min and then immediately for 1 h at room temperature, without intermediate washes. Formalin solution was removed, and cells were washed three times with PBS. Cells were incubated for 5 min at room temperature with 60% isopropanol and after complete drying, cells were incubated for 10 min with the Oil Red-O working solution (filtered 6:4 dilution of a pre-filtered 0.35% Oil Red-O stock solution in 100% isopropanol with di-deionized water). Cells were washed with di-deionized water and completely dried. In order to evaluate lipid droplets content spectrophotometrically, Oil Red-O incorporated in lipid droplets was eluted with a unique wash in 100% isopropanol for 10 min at room temperature by gentle shaking and relative absorbance measured at 510 nm with an Eppendorf BioSpectrometer®.

### Determination of mitochondrial mass and potential

In order to identify mitochondrial mass, cells were incubated with 200 nM MTG (Mito Tracker Green, Invitrogen) 30 min before the end of the experimental time, washed with PBS and re-suspended in PBS, and the fluorescence intensity immediately analyzed cytofluorometrically by recording FL-1 fluorescence by means of a FACScalibur instrument. 10,000 events were counted, and data expressed as arbitrary units. To verify the potential of mitochondria, cells were treated with 200 nM MTR (Mito Tracker Red, Invitrogen) for 30 min and it was followed the same protocol as that of MTG, analyzing samples through cytofluorimeter by recording FL-2.

### Extracellular lactate assay

Extracellular lactate assay was performed considering 500 μl of cell medium, that was precipitated with 250 μl of 30% trichloroacetic acid (TCA). Media were collected at − 20° for at least 1 h and then centrifuged at 14,000×g for 20 min at 4 °C. 10 μl of supernatant were incubated for 30 min at 37 °C in 290 μl of reaction buffer (0.2 M glycine, 0.2 M hydrazine buffer pH 9.2 with freshly added 0.6 mg/ml NAD^+^ and 17 U/ml LDH enzyme). After 30 min at 37 °C, LDH-mediated conversion of lactate into pyruvate with the reduction of NAD^+^ into NADH is completed. The reaction forms nmoles of NADH, stoichiometrically equivalent to extracellular lactate, allowing to assess the amount of lactate by measuring the amount of NADH at 340 nm using an Eppendorf BioSpectrometer®. Thus, relative absorbance values were converted to lactate concentration using an extinction coefficient of 6220 M^− 1^ cm^− 1^ at 340 nm for NADH. Concentrations were normalized on total proteins in the samples.

### ROS evaluation

Thirty min before the end of the experimental time, cells were incubated with 50 μM dihydroethidium (DHE) (Molecular probes, cat. Number D1168) at 37 °C. Cells were then washed and re-suspended in PBS. The fluorescence intensity of ethidium, formed by the reaction of DHE with ROS, was analyzed cytofluorometrically by recording FL-2 fluorescence. 10,000 events were counted for each sample, in which the amount of superoxide was quantified based on the shift of the fluorescence intensity of the cell population marked with dihydroethidium.

### Apoptosis assay

Cells transfected with ATGL and BNIP3 ΔTM plasmids were seeded in a 12-well plate. After 48 h of over-expression, the cells were collected and stained with an Annexin V-FITC/propidium iodide (PI) following manufacturer’s protocol of Annexin V FITC Assay kit (Cayman Chemical, cat. Number 600300). After an incubation for 10 min at room temperature in the dark, the apoptotic rate of the cells was detected by flow cytometry (CytoFLEX S, Beckman Coulter, USA). CytExpert software was used to analyze the results.

### Measurement of hexokinase (HK) activity

HK activity was spectrophotometrically determined. Cells were wash in PBS and lysed for 30 min on ice in lysis buffer (50 mM Tris-HCl, pH 7.5, 1 mM EDTA, 150 mM NaCl, 1% NP-40, 1 mM DTT, protease inhibitor cocktail). The optical absorbance was measured at 340 nm every 15 s for 10 min with an Eppendorf BioSpectrometer® after incubation of 50 μg of protein lysate in reaction buffer (50 mM Tris-HCl, pH 7.5, 10 mM MgCl2, 0.6 mM ATP, 100 mM glucose, 0.2 mM NADP+, 0.1 U/mL of glucose-6-phosphate dehydrogenase) for 30 min at 37 °C. Enzyme activity was represented as change in absorbance per minute (U), normalized on protein.

### Bioinformatic analyses

*PNPLA2* expression in the different grades of cervical cancer was assessed by Gene Expression Omnibus (GEO; http://www.ncbi.nlm.nih.gov/geo, accession number GSE63514) through an Affymetrix Human Genome Array (100 samples of cervical cancer subdivided for grade vs normal cervical epithelium; normal = 24, low grade = 14, moderate grade = 22, high grade = 40).

### Data analysis

Data were from at least 3 independent experiments. The results are presented as means ± SD. Statistical evaluation was conducted by the Student *t-*test for comparison of only two variables and one-way ANOVA with post hoc Tukey for multiple comparisons, by using the GraphPad Prism 7 software. Comparisons were statistically considered significant at *p* ≤ 0.05 (*), very statistically significant at *p* ≤ 0.01 (**) and extremely statistically significant at *p* ≤ 0.001 (***). Statistics are reported in every figure and were determined according to published literature.

## Results

### ATGL over-expression is associated with an increased proliferation rate in cervical cancer cell lines

**Fig. 1:**
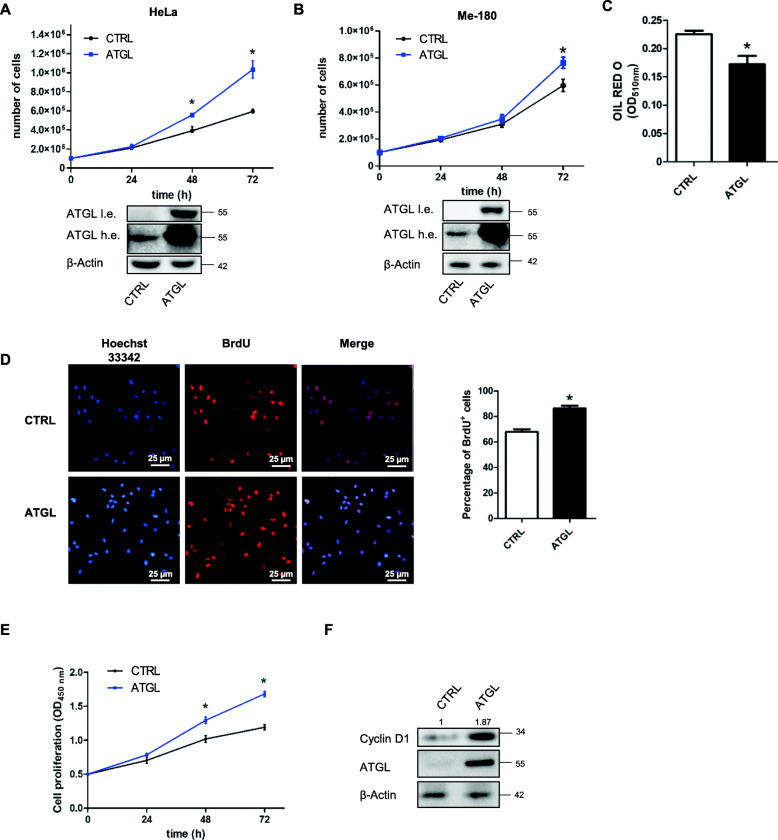
ATGL over-expression is associated with increased proliferation rate in cervical cancer cell lines. (**a**) HeLa and (**b**) Me-180 cells were transfected with ATGL plasmid and after 24, 48 and 72 h the proliferation was assayed by Trypan blue direct cell counting procedure. Data are expressed as mean ± SD (*n* = 3; * *p < 0.05* vs. CTRL). Bottom panels showed the Western blot analysis of the transfection efficiency. The high exposure (h.e.) allows to show ATGL expression in the CTRL sample. β-Actin was used as loading control. HeLa cells were transfected with ATGL plasmid for 48 h and (**c**) spectrophotometric determination of lipid droplets content after Oil Red-O staining was assessed. Oil Red-O incorporated in lipid droplets was eluted in isopropanol for 10 min and relative absorbance measured at 510 nm was normalized on total proteins. Data are expressed as mean ± SD (*n* = 3; * *p < 0.05* vs. CTRL). (**d**) BrdU incorporation was quantified in HeLa cells by immunofluorescence, after 24 h of 10 μM BrdU treatment. Images reported are representative of three independent experiments. Nuclei were stained 10 min with 1 μg/mL Hoechst 33342. Bar graph refers to the percentage of BrdU-positive cells. Data are expressed as mean ± SD (*n* = 3; * p < 0.05 vs. CTRL). (**e**) Proliferation assayed by CCK-8 colorimetric assay. Data are expressed as mean ± SD (n = 3; * *p < 0.05* vs. CTRL). (**f**) The levels of cell cycle regulator Cyclin D1 were evaluated by Western blot analysis. Band intensity is indicated below the corresponding band and expressed as fold-change relative to CTRL. The images are representative of three independent experiments that gave similar results. β-Actin and ATGL were used as loading and transfection control, respectively

To investigate the role of intracellular lipolysis and lipid metabolism in cervical cancer, we over-expressed the lipase ATGL and we assessed whether the proliferative capacity of cervical cancer cells was affected. Figures 1a and b showed an increased proliferation rate of both HeLa and Me-180 cervical cancer cell lines as determined by Trypan blue exclusion assay. The decrement in the stored intracellular lipid content of HeLa cells, measured by Oil Red O staining, proved that the transfected ATGL protein was active (Fig. 1c). We focused on HeLa cells, in which ATGL affected proliferation more evidently, and confirmed by bromodeoxyuridine (BrdU) incorporation assay (Fig. 1d) and CCK-8 assay (Fig. 1e) the proliferation increase upon ATGL over-expression. Moreover, Cyclin D1, which drives the G1/S phase transition, resulted higher upon ATGL over-expression (Fig. 1f).

### Increased ATGL levels induce an aggressive phenotype in HeLa cells

**Fig. 2:**
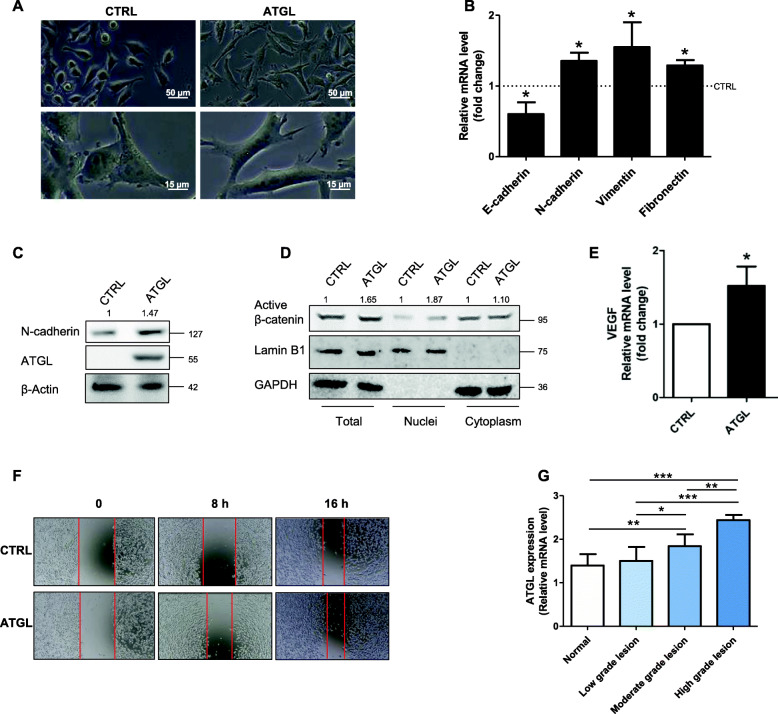
Increased ATGL levels induce an aggressive phenotype in HeLa cells. HeLa cells were transfected with pATGL for 48 h. (**a**) Morphological change of HeLa cells transfected with pcDNA™4/HisMaxC and pcDNA™4/HisMaxC-ATGL for 48 h. (**b**) RT-qPCR analysis of E-cadherin, N-cadherin, Vimentin and Fibronectin mRNA. β-Actin (*ACTB*) was used as reference control. Data are shown as fold change vs. CTRL represented by a dashed line in the bar graph (*n* = 4; * *p* < 0.05 vs. CTRL). (**c**) Western blot analysis of mesenchymal marker N-cadherin levels. β-Actin and ATGL were used as loading and transfection control, respectively. Band intensity is indicated below the corresponding band and expressed as fold-change relative to CTRL. The Western blots reported are representative of three independent experiments that gave similar results. (**d**) Western blot analysis of active β-catenin levels on nuclear extracts. Lamin B1 and Glyceraldehyde 3-phosphate dehydrogenase (GAPDH) were used as nuclear and cytosolic purity control, respectively. Band intensity is indicated below the corresponding band and expressed as fold-change relative to CTRL for each fraction. The Western blots reported are representative of three independent experiments that gave similar results. (**e**) RT-qPCR analysis of VEGF mRNA. *ACTB* was used as reference control. Data are showed as fold change vs. CTRL (n = 4; * *p* < 0.05 vs. CTRL). (**f**) Wound Healing assay. HeLa cells were seeded onto 12-well plates and transfected with pATGL for 48 h. The monolayer was wounded with a plastic tip and monitored under bright-field microscope, taking images at 0, 8, and 16 h from “wound” formation; images, representative of three independent experiments that gave similar results. (**g**) *Patatin Like Phospholipase Domain Containing 2* (*PNPLA2)* gene expression in different grade of cervical cancer was assessed by Gene Expression Omnibus (GEO; http://www.ncbi.nlm.nih.gov/geo, accession number GSE63514) through an Affymetrix Human Genome Array (100 samples of cervical cancer subdivided for grade vs. normal cervical epithelium) (* *p < 0.05;* ** *p < 0.01;* *** *p < 0.001* as indicated)

To gain further insight into the pro-tumoral activity of ATGL, we assessed whether its over-expression had any effects on cervical cancer aggressiveness. Firstly, we observed that ATGL induced significant changes in cell morphology: as shown in Fig. 2a, HeLa cells become elongated after ATGL over-expression (Fig. 2a). Therefore, we analyzed the epithelial/mesenchymal transition (EMT) process by measuring the expression levels of the well-known mesenchymal markers N-cadherin, Vimentin and Fibronectin. As shown in Fig. 2b, ATGL over-expression significantly increased the transcription of all those genes with respect to control cells. Furthermore, protein levels of N-cadherin were positively associated with ATGL expression (Fig. 2c). On the other side, the expression of the epithelial marker E-cadherin was downregulated in ATGL-overexpressing cells (Fig. 2b). In support of the hypothesis that lipolysis promotes the aggressive phenotype of HeLa cells, over-expression of ATGL was associated with increased nuclear β-catenin (Fig. 2d), a marker of cancer cell invasion [24, 25] and the up-regulation of its known target and angiogenesis factor Vascular endothelial growth factor (VEGF) [26] (Fig. 2e). ATGL over-expressing cells also showed an increased migration capacity, as verified by Wound Healing assay (Fig. 2f). Finally, to support our results with in vivo evidence, we performed bioinformatic analysis of the Affymetrix-U133-plus2.0 array on a large dataset of human cervical cancer samples subdivided for grade vs. normal cervical epithelium. The data reported in Fig. 2g showed that in cervical cancer the expression levels of ATGL were higher in tumor samples and positively associated with tumor grade, confirming a pro-tumoral role for the lipase.

### ATGL over-expression promotes the “Warburg effect” in HeLa cells

**Fig. 3:**
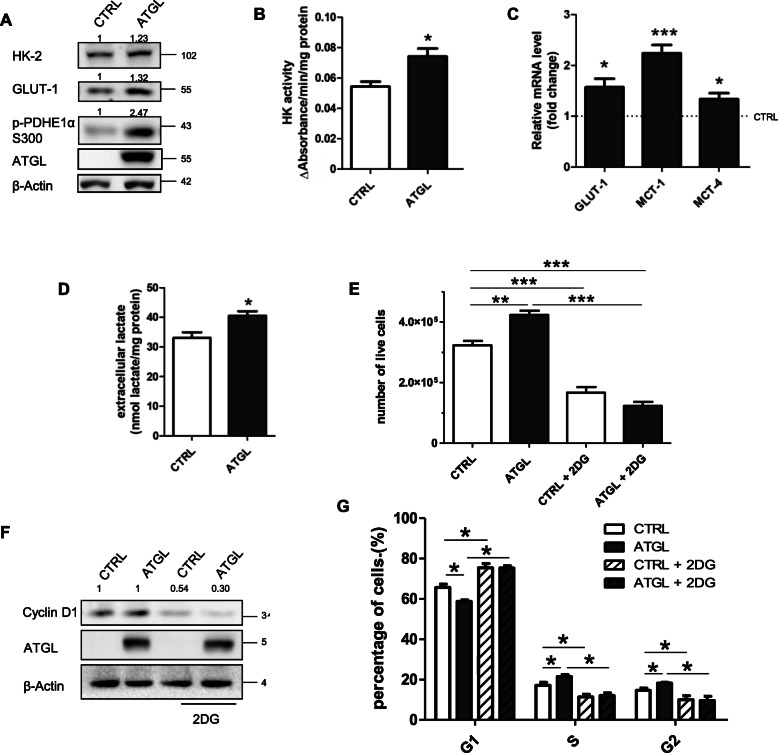
ATGL over-expression promotes the “Warburg effect” in HeLa cells. HeLa cells were transfected for 48 h with pATGL. HeLa cells were over-expressed for 48 h and glycolytic metabolism was monitored by: (**a**) Western blot analysis of hexokinase-2 (HK-2), glucose transporter 1 (GLUT1) and phosphorylated pyruvate dehydrogenase (p-PDHE1α S300) levels. Band intensity is indicated below the corresponding band and expressed as fold-change relative to CTRL. The images are representative of three independent experiments that gave similar results. β-Actin and ATGL were used as loading and transfection control, respectively; (**b**) spectrophotometric determination of hexokinase (HK) activity. Relative absorbance was normalized on total proteins and data are expressed as mean ± SD (*n* = 3; * *p < 0.05* vs. CTRL); (**c**) the expression levels of glucose and lactate transporters genes through RT-qPCR analysis of *GLUT1*, *MCT1* and *MCT4*. *ACTB* was used as reference control. Data are shown as fold change vs. CTRL represented by a dashed line in the bar graph (n = 3; * *p < 0.05* vs. CTRL); (**d**) Evaluation of extracellular lactate content was performed on cell culture media collected after 48 h of ATGL over-expression. Relative absorbance was normalized on total proteins and data are expressed as mean ± SD (n = 3; * *p < 0.05* vs. CTRL). Cells were treated with the glycolysis inhibitor 2-deoxyglucose (2DG, 10 mM) 24 h before the end of the experiment upon ATGL over-expression and (**e**) proliferation rate was assayed by Trypan blue direct cell counting procedure. Data are expressed as mean ± SD (n = 3; ** *p < 0.01;* *** *p < 0.001* as indicated). (**f**) Cyclin D1 levels were analyzed by Western blot analysis. Band intensity is indicated below the corresponding band and expressed as fold-change relative to the untreated condition. The images are representative of three independent experiments that gave similar results. β-Actin and ATGL were used as loading and transfection control, respectively. (**g**) Statistical analysis of HeLa cells percentage in the different phases of the cell cycle after 48 h of ATGL over-expression determined by FACS analysis. The values represent the number of cells in each phase of the cell cycle as a percentage of the total cells. Data are expressed as mean ± SD (n = 3; * *p < 0.05* as indicated)

The proliferative advantage of cancer cells often depends on increased glycolytic rate, therefore, we verified whether this phenomenon also occurred upon ATGL over-expression by measuring protein levels of the glucose transporter 1 (GLUT1) and hexokinase-2 (HK-2), the first and rate-limiting enzyme of glycolysis. We revealed an increase in uptake and utilization of glucose (Fig. 3a) and a higher hexokinase activity assessed by spectrophotometric analysis (Fig. 3b). The transcriptional upregulation of GLUT1 induced by ATGL over-expression was paralleled by increased mRNA levels of the lactate transporters Monocarboxylate transporter 1 (MCT1) and Monocarboxylate transporter 4 (MCT4) (Fig. 3c). The increase in extracellular lactate content (Fig. 3d) and the inhibitory phosphorylation of pyruvate dehydrogenase (p-PDHEiα S300) (Fig. 3a), which is the enzyme that promotes glucose oxidation in the Krebs cycle converting pyruvate to acetyl-CoA, depicted a scenario in which the downstream glycolytic product pyruvate was mainly reduced to lactate and extruded by the cell, rather than directed towards mitochondria. Overall, these results indicated that ATGL over-expression was accompanied by an enhancement of the “Warburg effect”. To substantiate the association of increased proliferation in our conditions with the glycolytic rate, we treated cells with the glycolysis inhibitor 2-deoxyglucose (2DG, 10 mM) for 24 h. The data reported in Fig. 3e indicated that although 2DG negatively affected proliferation in both control and ATGL over-expressing cells, as also confirmed by Cyclin D1 levels (Fig. 3f) and by the cell cycle block at G1 phase (Fig. 3g), it abrogated the proliferative advantage of cells with increased levels of ATGL, demonstrating that it was dependent on the glycolytic metabolism.

### BNIP3-mediated mitophagy contributes to the increased proliferation rate as a pro-survival cell response

**Fig. 4:**
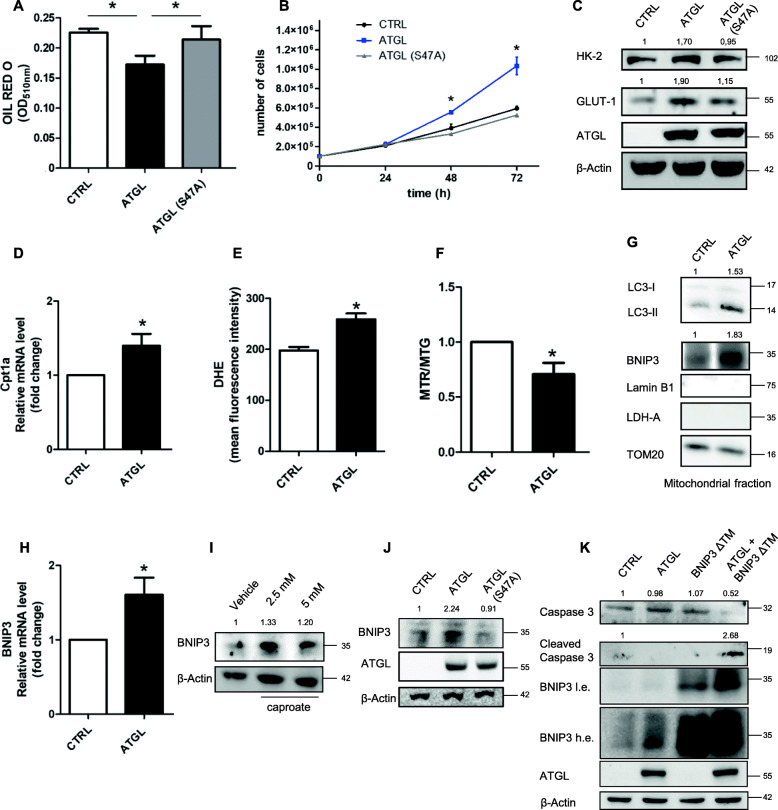
BNIP3-mediated mitophagy contributes to the increased proliferation rate as a pro-survival cell response. HeLa cells were transfected with ATGL and ATGL-S47A plasmids for 48 h. (**a**) Spectrophotometric determination of lipid droplets content after OIL red O staining. Relative absorbance was normalized on total proteins. Data are expressed as mean ± SD (*n* = 3; * *p < 0.05* as indicated). (**b**) Proliferation was assayed by Trypan blue direct cell counting procedure. Data are expressed as mean ± SD (n = 3; * *p < 0.05* vs. CTRL and ATGL-S47A). (**c**) HK-2 and GLUT1 levels were evaluated by Western blot analysis. Band intensity is indicated below the corresponding band and expressed as fold-change relative to CTRL. Images are representative of three independent experiments that gave similar results. β-Actin and ATGL were used as loading and transfection control, respectively. (**d**) RT-qPCR analysis of Cpt1a mRNA. *ACTB* was used as reference control. Data are showed as fold change vs. CTRL (*n* = 3; * *p < 0.05* vs. CTRL). (**e**) Reactive Oxygen Species (ROS) were quantified by cytofluorimetric analysis. Cells were treated with 50 μM of DHE 30 min before the end of the experiment. 10,000 events were counted. Data are expressed as mean ± SD (n = 3; * *p < 0.05* vs. CTRL). (**F**) Ratio between the mean fluorescence intensity of MTR, which measures the mitochondrial potential, and of MTG, which measures the mitochondrial mass. Cytofluorimetric analysis was performed after treatment with 200 μM of MTR and MTG 30 min before the end of the experiments. 10,000 events were counted. Data are expressed as mean ± SD (n = 3; * *p < 0.05* vs. CTRL). (**g**) Western blot analysis on mitochondrial fraction of LC3 and BNIP3 levels. Band intensity is indicated below the corresponding band and expressed as fold-change relative to CTRL. Images are representative of three independent experiments that gave similar results. Lamin B1 and Lactate dehydrogenase A (LDH-A) were used as mitochondrial purity controls. TOM20 was used as loading control. (**h**) RT-qPCR analysis of BNIP3 mRNA. *ACTB* was used as reference control. Data are showed as fold change vs. CTRL (n = 3; * *p < 0.05* vs. CTRL). (**i**) Western blot analysis of BNIP3 levels after treatment of HeLa cells with 2.5 mM and 5 mM caproate for 24 h. Band intensity is indicated below the corresponding band and expressed as fold-change relative to vehicle. Images are representative of three independent experiments that gave similar results. β-Actin was used as loading control. (**j**) After over-expression of ATGL and ATGL-S47A (catalytic mutant), BNIP3 levels were evaluated by Western blot analysis. Band intensity is indicated below the corresponding band and expressed as fold-change relative to CTRL. Images are representative of three independent experiments that gave similar results. β-Actin and ATGL were used as loading and transfection control, respectively. HeLa cells were transfected with pATGL and BNIP3 ΔTM plasmid for 48 h and (**k**) Western blot analysis of cleaved caspase 3 was assayed. Band intensity is indicated below the corresponding band and expressed as fold-change relative to CTRL. Images are representative of three independent experiments that gave similar results. β-Actin was used as loading and transfection control; ATGL and BNIP3 were used as transfection controls. The high exposure (h.e.) of BNIP3 allows to show the protein levels in CTRL and ATGL samples

We next evaluated the possibility that the increased FAs utilization mediated the higher proliferation of ATGL-overexpressing HeLa cells and the associated anaerobic pathway of glucose. To this aim, we took advantage of a catalytic inactive mutant of ATGL, characterized by a substitution of the serine-47 of the catalytic dyad with an alanine (ATGL-S47A). Unlike wild-type ATGL, the over-expression of the inactive isoform of ATGL did not affect the content of neutral lipids (Fig. 4a). Consistently, ATGL-S47A over-expression did not affect cell proliferation, confirming that the catalytic activity of ATGL is required for this process (Fig. 4b). Similarly, ATGL activity was also required for the higher glycolytic rate of ATGL over-expressing cells, as shown by the levels of GLUT1 and HK-2 that were not upregulated in the presence of ATGL-S47A (Fig. 4c). To associate these effects with FAs utilization inside mitochondria, we measured the levels of Carnitine Palmitoyltransferase 1a (Cpt1a), the enzyme that controls the mitochondrial uptake of long-chain fatty acids typically stored in LDs and determines β-oxidation flux (Fig. 4d). The increased levels of Cpt1a in ATGL-overexpressing cells suggested that mitochondrial lipid catabolism was enhanced, as also corroborated by the accumulation of ROS (Fig. 4e). To verify if the increase of ROS levels deriving from forced FAs utilization had any effect on mitochondria functionality we performed a cytofluorimetric analysis using MitoTracker Red (MTR) and MitoTracker Green (MTG) probes: ATGL over-expressing cells displayed a lower mitochondrial membrane potential suggesting that mitochondria were less functional (Fig. 4f). Considering mitophagy as the main cellular mechanism to degrade defective mitochondria, we evaluated the levels of the mitochondrial proteins Translocase of outer mitochondrial membrane 20 (TOM20) and Mitochondrial transcription factor A (TFAM) after treatment with the autophagy inhibitor chloroquine (Cq). The accumulation of both proteins by Cq in ATGL-overexpressing cells (Supplementary Fig. S1A) and the stimulatory phosphorylation on serine-616 of dynamin-related protein 1 (DRP1) (Supplementary Fig. S1B), a marker of mitochondrial fission that is known to precede mitophagy, demonstrated the activation of the mitophagic process in our conditions. As HeLa cells do not express Parkin, we focused on BNIP3 as the mediator of mitophagy. Consistently, we observed an increase in BNIP3 levels together with the autophagosome marker Microtubule-associated protein 1A/1B-light chain 3 (LC3) in mitochondrial fraction of ATGL transfected cells (Fig. 4g) and their further accumulation after Cq treatment in total lysates (Supplementary Fig. S1C). RT-qPCR demonstrated that BNIP3 was transcriptionally induced in ATGL-overexpressing cells (Fig. 4h). Mitophagy activation could be a consequence of ATGL-mediated increase of lipid catabolism, which in turn enhances mitochondrial oxidative metabolism. We verified this hypothesis by mimicking ATGL activity using caproate, a short-chain fatty acid that easily crosses the mitochondrial membrane. Caproate resulted in BNIP3 induction (Fig. 4i) as observed after ATGL over-expression, whereas the use of ATGL-S47A was ineffective (Fig. 4j). BNIP3-mediated mitophagy could be engaged to mitigate cell damage and death due to ROS bursting under forced lipid catabolism. Thus, we investigated the effect of BNIP3 inhibition on cell proliferation and vitality. The transfection of the dominant-negative mutant of BNIP3 lacking the C-terminal transmembrane domain (BNIP3 ΔTM) decreased the number of live cells over-expressing ATGL (Supplementary Fig. S2A) and induced a high amount of cleaved-caspase 3 (Fig. 4k), indicative of the commitment of cells to apoptosis, that we observed by staining cells with annexin V and PI (Supplementary Fig. S2B). These results suggested that BNIP3-induced mitophagy was required for promoting cell survival and proliferation rate upon the increase of oxidative metabolism due to lipid utilization.

### ATGL activity mediates the activation of HIF1α, responsible for mitophagy and glycolysis induction

**Fig. 5:**
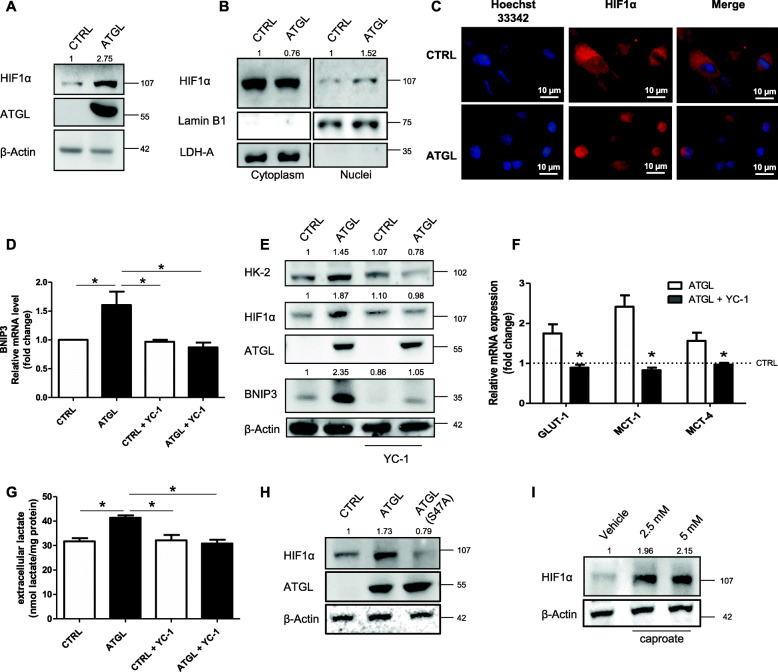
ATGL activity mediates the activation of HIF1α, responsible for mitophagy and glycolysis induction. HeLa cells were transfected with ATGL plasmid for 48 h. (**a**) Western blot analysis of HIF1α levels. β-Actin and ATGL were used as loading and transfection control, respectively. Band intensity is indicated below the corresponding band and expressed as fold-change relative to CTRL. Images are representative of six independent experiments that gave similar results. (**b**) Western blot analysis of HIF1α levels on nuclear extracts. Lamin B1 and LDH-A were used as nuclear and cytosolic purity control, respectively. Band intensity is indicated below the corresponding band and expressed as fold-change relative to CTRL for each fraction. Images are representative of three independent experiments that gave similar results. (**c**) Immunofluorescent analysis of HIF1α subcellular localization in HeLa cells after ATGL over-expression. Images are representative of three independent experiments that gave similar results. Nuclei were stained 10 min with 1 μg/mL Hoechst 33342. HeLa cells were transfected with ATGL plasmid for 48 h and were treated with the HIF1α inhibitor (YC-1, 100 μM) 24 h before the end of the experiment. (**d**) RT-qPCR analysis of BNIP3 mRNA was performed. *ACTB* was used as reference control. Data are shown as fold change (*n* = 3; * *p < 0.05* as indicated). Glycolytic metabolism was monitored by: (**e**) Western blot analysis of HK-2 levels. β-Actin and ATGL were used as loading and transfection control, respectively; HIF1α was evaluated as YC-1 treatment control. Band intensity is indicated below the corresponding band and expressed as fold-change relative to CTRL for each fraction. Images are representative of three independent experiments that gave similar results; by (**f**) RT-qPCR analysis of *GLUT1, MCT1* and *MCT4* mRNA. *ACTB* was used as reference control. Data are shown as fold change (n = 3; * *p < 0.05* vs ATGL). CTRL was represented by a dashed line in the bar graph. (**g**) Evaluation of extracellular lactate content, relative absorbance was normalized on total proteins. Data are expressed as mean ± SD (n = 3; * *p < 0.05;* ** *p < 0.01* as indicated); (**h**) After over-expression of ATGL and ATGL-S47A (catalytic mutant) HIF1α levels were evaluated by Western blot analysis. Band intensity is indicated below the corresponding band and expressed as fold-change relative to CTRL. Images are representative of three independent experiments that gave similar results. β-Actin and ATGL were used as loading and transfection control, respectively. (**I**) Western blot analysis of HIF1α levels performed for HeLa cells treated with 2.5 mM and 5 mM caproate for 24 h. Band intensity is indicated below the corresponding band and expressed as fold-change relative to Vehicle. Images are representative of three independent experiments that gave similar results. β-Actin was used as loading control

Among the transcription factors activating BNIP3 expression the most characterized is HIF1α [27], which attracted our attention because it is also known for promoting glycolysis and proliferation of cervical cancer cells [28]. In our system, HIF1α was positively associated with ATGL levels (Fig. 5a) and more present in nuclei of cells transfected with ATGL (Fig. 5b, c). Using the HIF1α inhibitor YC-1 in HeLa cells, we demonstrated that BNIP3 induction was abolished at both mRNA (Fig. 5d) and protein level (Fig. 5e). ATGL-mediated activation of HIF1α-BNIP3 axis was also confirmed in Me-180 cells (Supplementary Fig. S3A). Beyond BNIP3, we observed that the higher glycolytic flux occurring after ATGL over-expression was due to HIF1α upregulation as the treatment with YC-1 diminished HK-2 (Fig. 5e), GLUT1, MCT1 and MCT4 expression levels (Fig. 5f) as well as extracellular lactate release (Fig. 5g). To link the increased HIF1α level to the enhancement of lipid catabolism, we demonstrated that the over-expression of ATGL catalytic mutant did not induce HIF1α expression (Fig. 5h) whereas this occurred after treatment with the short-chain fatty acid caproate (Fig. 5i).

### ROS are the upstream mediators of the pseudo-hypoxic response occurring after ATGL over-expression

**Fig. 6:**
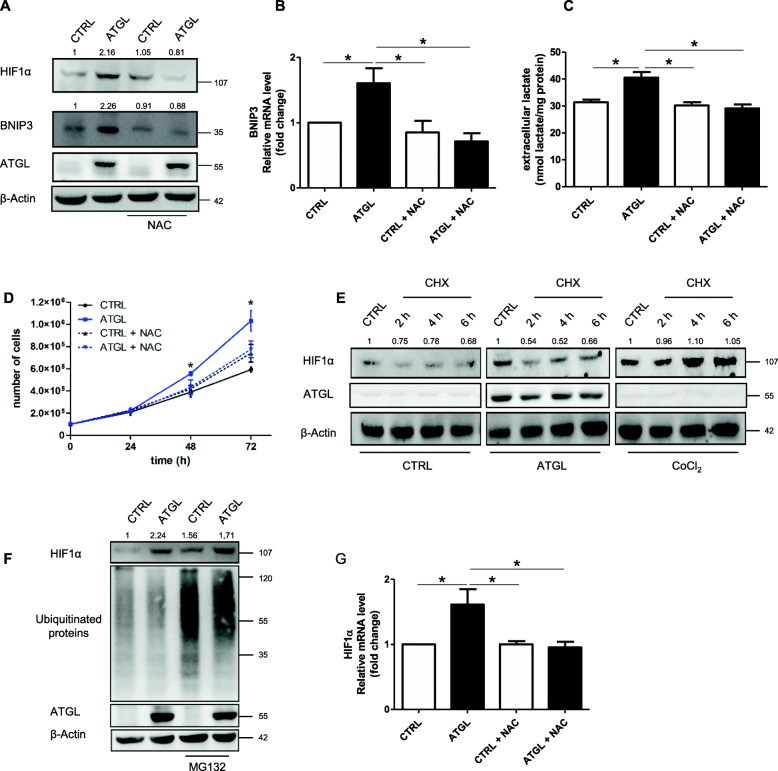
ROS are the upstream mediators of the pseudo-hypoxic response occurring after ATGL overexpression. HeLa cells were transfected with ATGL plasmid for 48 h and treated with 5 mM NAC 24 h before the end of the experiment. (**a**) HIF1α and BNIP3 levels were analyzed by Western blot analysis. Band intensity is indicated below the corresponding band and expressed as fold-change relative to CTRL. The images are representative of three independent experiments that gave similar results. β-Actin and ATGL were used as loading and transfection control, respectively. (**b**) RT-qPCR analysis of BNIP3 levels. *ACTB* was used as reference control. Data are shown as fold change (n = 3; * *p < 0.05* as indicated). (**c**) The evaluation of extracellular lactate content was performed on cell culture media collected after 48 h of ATGL over-expression by spectrophotometric analysis. Data are expressed as mean ± SD (n = 3; * *p < 0.05* as indicated). (**d**) The proliferation was assayed by Trypan blue direct cell counting procedure. Data are expressed as mean ± SD (n = 3; * *p < 0.05* vs. ATGL + NAC). (**e**) Cells were treated with Cycloheximide (CHX, 10 μg/mL) for 2, 4, 6 h and cobalt chloride (CoCl_2_, 150 μM) for 6 h before the end of experiment and HIF1α protein levels were evaluated by Western blot analysis. Band intensity is indicated below the corresponding band and expressed as fold-change relative to CTRL for each condition. The images are representative of three independent experiments that gave similar results. β-Actin and ATGL were used as loading and transfection control, respectively. (**f**) HeLa cells were treated with MG132 (2 μM) for 8 h before the end of the experiment and Western blot analysis of HIF1α and ubiquitinated proteins was determined. Band intensity is indicated below the corresponding band and expressed as fold-change relative to CTRL. The images are representative of three independent experiments that gave similar results. β-Actin and ATGL were used as loading and transfection control, respectively. HeLa cells were transfected as previously described and were treated with 5 mM NAC 24 h before the end of the experiment and (**g**) RT-qPCR analysis of HIF1α levels was performed. *ACTB* was used as reference control. Data are shown as fold change (n = 3; * *p < 0.05* as indicated)

Considering that ATGL over-expression determined an increase in ROS production (Fig. 4e) and that HIF1α can be induced by ROS [17], we recapitulated the main experiments concerning mitophagy, glycolysis and cell proliferation upon antioxidants treatment. We demonstrated that following N-acetylcysteine (NAC) treatment, ATGL over-expression did not affect HIF1α and BNIP3 protein levels, in both HeLa cells (Fig. 6a) and Me-180 cells (Supplementary Fig. S3B). Furthermore, NAC was also efficient in abrogating the increased BNIP3 mRNA induction as a consequence of the HIF1α decrement (Fig. 6b). As for glycolysis, it returned at the control level after NAC treatment, as shown by the data of extracellular lactate content (Fig. 6c), and consequently also the increase in the proliferation rate was abolished (Fig. 6d).

It is known from the literature that ROS can regulate HIF1α in different ways, by inducing transcription, translation or protein stabilization [17, 29]. To discriminate between transcriptional and post-translational regulation of HIF1α in our system, we treated HeLa cells with cycloheximide (CHX) to block de novo protein synthesis, or the proteasome inhibitor MG132 to block protein degradation. HIF1α was not induced upon CHX treatment in ATGL over-expressing cells, supporting that its increase depends on mRNA translation (Fig. 6e). On the contrary, CHX treatment did not affect HIF1α protein levels when combined with CoCl_2_ treatment, a canonical hypoxia-mimetic agent known to stabilize HIF1α protein (Fig. 6e). Moreover, the increase in HIF1α levels upon MG132 treatment in ATGL over-expressing cells (Fig. 6f) mirrored the one observed in control cells suggesting the maintenance of a similar turnover of HIF1α protein in both conditions. In support of the hypothesis that HIF1α was transcriptionally regulated, we observed increased HIF1α mRNA level following ATGL over-expression, whereas NAC treatment reverted this effect proving the causative role of ROS in this process (Fig. 6g).

## Discussion

Lipid metabolism is part of the metabolic rewiring occurring in tumorigenesis and tumor metastasis [30]. Here, we dissected the effects of boosting lipid catabolism through ATGL over-expression in cervical cancer cells. Using HeLa and Me-180 cell lines we showed that ATGL over-expression promoted cervical cancer cells proliferation demonstrating a pro-tumor feature of ATGL that was also observed in prostate cancer, pancreatic ductal adenocarcinoma, and breast cancer [31–33]. The switch from a proliferative to a migratory state needs the selective release of specific FAs that can function as signalling molecules (mainly deriving from membrane lipids) and as constituents for membranes that are remodeled to facilitate changes in fluidity and thus cell migration (mainly intracellular lipids) [34]. Indeed, supplementation with FAs increases cell migration [35]. Moreover, the lipolytic lipase MAGL was extensively associated with the EMT phenotype and aggressiveness in several tumor types [10, 36]. Our data on EMT highlighted that also ATGL upregulation exacerbated the aggressiveness of HeLa cells promoting cell morphology changes, the expression of mesenchymal and invasion markers and migratory capacity. In line with this, the data from a publicly available microarray database show that the expression of ATGL positively correlates with the aggressiveness of cervical cancer. In our system, the increased proliferation of ATGL over-expressing cells was dependent on a higher glycolytic rate, as the proliferative advantage was lost when they were treated with the glycolysis inhibitor 2DG. The enhancement of glycolysis was determined through the increase of GLUT1 expression and of the level/activity of HK-2, the first check-point enzyme of the glycolytic pathway. The high activity of HK-2 is a typical feature of malignant cells and is responsible for the accelerated glycolytic metabolism contributing to tumorigenesis and metastasis [37]. The sustained glycolytic flux in ATGL-overexpressing cells culminated in the exacerbation of the “Warburg effect” as demonstrated by the upregulation of the lactate transporters MCT1/MCT4 and the higher concentration of extracellular lactate.

The phenotypic effects we observed were dependent on the metabolic adaptation driven by FAs availability that could feed the Krebs cycle. Indeed, FAs oxidation is accompanied by inhibitory phosphorylation of PDH [38], the enzyme that catalyzes the aerobic conversion of pyruvate to acetyl-CoA, and that increased after ATGL over-expression thus contributing to diverge glucose toward the “Warburg effect”. The importance of FAs was validated by the use of ATGL catalytic mutant that did not alter the proliferation rate of HeLa cells, nor it causes the glycolysis-related changes. The increase in FAs oxidation was supported by the upregulation of the mitochondrial long-chain fatty acids transporter Cpt1a, which couples acyl chains to carnitine for transport into the mitochondrial matrix and subsequent degradation. FAs oxidation is usually associated with increased ROS production that could be detrimental for cells if not adequately buffered. This is particularly true for cancer cells, normally characterized by higher levels of intracellular ROS [12, 39]. ROS increase occurring downstream of ATGL-mediated FAs release was harmful to mitochondria and affected mitochondrial potential. Cells coped with oxidative damage by upregulating the removal of dysfunctional mitochondria by mitophagy. Mitophagy has frequently been demonstrated to mitigate oxidative stress deriving from enhanced mitochondrial oxidative metabolism during cancer metabolic adaptations [40, 41]. We have identified BNIP3 as the mediator of mitophagy in our conditions. We excluded this is only the consequence of the absence of Parkin in HeLa cells as BNIP3 was also upregulated in Me-180 cells after ATGL over-expression [42]. Caproate, a short-chain fatty acid that is easily oxidized into mitochondria, was sufficient to induce BNIP3, whereas the ATGL catalytic mutant failed, confirming that BNIP3 was transcriptionally induced in response to increased lipid catabolism. In support of the connection between lipid catabolism and BNIP3-mediated mitophagy, there is also in vivo evidence of decreased β-oxidation and accumulation of LDs in BNIP3 null mice [43]. Inhibition of BNIP3 through its dominant-negative mutant reduced proliferation and increased apoptotic cell death in ATGL over-expressing cells, highlighting the key role of the BNIP3-mediated mitophagy as a pro-survival event. The findings so far discussed unveil two main effects of boosting lipid catabolism in HeLa cells: the induction of glycolysis and the promotion of mitophagy as mediators of increased proliferation. The activation of HIF1α was responsible for this phenotype. Indeed, HIF1α is known to promote the metabolic switch towards glycolysis in the hypoxic condition, when OXPHOS is inefficient, as well as the upregulation of BNIP3 by binding the hypoxia response elements (HRE) on BNIP3 promoter [44, 45]. Both the promotion of anaerobic production of ATP and the degradation of mitochondria, which are the main consumers of cellular oxygen and the primary source of ROS, allow the metabolic adaptation to oxygen deprivation and exacerbate the malignancy of cancer cells [46]. However, the activation of HIF1α occurs frequently in normoxia conditions, in an oxygen-independent manner, a phenomenon defined as pseudo-hypoxia [47]. ATGL mediated pseudo-hypoxic response is mimicked by caproate treatment, while ATGL catalytic mutant failed to activate HIF1α, supporting the involvement of the enhanced lipolysis in the induction of HIF1α. HIF1α activation and the association with the “Warburg effect” were also demonstrated in prostate cancer cells that receive lipids from marrow adipocytes [48].

The enhanced ROS production, which is known to be involved in HIF1α activation [49] is the event upstream of the pseudo-hypoxic response. Indeed, several studies demonstrated the association between abundant ROS generation by mitochondria and HIF1α induction. Moreover, ROS can trigger cancer metabolic changes (e.g.*,* “Warburg effect”) aimed to reduce mitochondrial oxidative stress and to prolong cancer cell viability. Despite several pieces of evidence showed that ROS can stabilize HIF1α protein even in normoxia, ROS-mediated transcriptional up-regulation of HIF1α can also occur [47]. We showed that upon ATGL over-expression HIF1α increase was reverted by inhibition of protein synthesis, suggesting that the translation of HIF1α mRNA, higher in ATGL transfected cells, was necessary to observe the protein increase. Future studies will be dedicated to investigating the molecular events behind this result focusing both on redox-dependent transcription factors and the mTOR/p70S6K/RPS6 signaling pathway known to promote HIF1α mRNA translation [50]. Moreover, the use of additional cell lines is necessary to broaden the significance of ATGL expression in cervical cancer aggressivness and to clarify whether the different effects of ATGL function are cancer-specific as a consequence of the antioxidant equipment or the intracellular LDs content of the cell. Indeed, along with evidence of ATGL tumor-promoting functions in this and other tumors, the antineoplastic role of ATGL was even demonstrated [2, 51]. We described that increased levels of ATGL in hepatocellular carcinoma cell lines diminished cell proliferation. In that cellular context, we did not observe any increase in ROS levels following ATGL over-expression even though the oxidative metabolism was efficiently activated. It is possible that the prominent antioxidant capacity of liver cells compared to others, like cervical cancer cells, allows them to efficiently face oxidative stress deriving from enhanced lipid catabolism, providing a rationale for the tumor-specific effect of ATGL.

## Conclusions

Overall, we propose a pro-tumor role of ATGL that is dependent on ROS which could be exploited for creating new personalized therapy based on the specific antioxidant system of tumor cells. Interestingly, HIF1α represents a key orchestrator of radioresistance [52] and paclitaxel resistance of cervical cancer [53]: even if these therapeutic approaches have various mechanisms of action, they all lead to an increase in ROS production. Indeed, the different profile of redox regulation systems of cervical cancer cells contributes to their response to treatment [54]. For this reason, the link here described between ATGL and HIF1α signaling mediated by ROS exposes a potential intervention point aimed to restrain cell resistance of cervical cancer cells.

## Supplementary Information

**Additional file 1 Fig. S1.** HeLa cells were transfected with ATGL plasmid for 48 h. Cells were treated with the autophagy inhibitor chloroquine (Cq, 30 μM) for 2 h and (**A**) Western blot analysis of mitochondrial markers TFAM and TOM20 was performed. Bar graph refers to the densitometry TOM20/β-Actin and TFAM/β-Actin ratio. The images are representative of three independent experiments that gave similar results. Data are shown as fold change (*n* = 3; * *p < 0.05;* *** *p < 0.001* as indicated). β-Actin and ATGL were used as loading and transfection control, respectively. (**B**) Western blot analysis of phosphorylated DRP1 (p-DRP1 S616) and DRP1 levels, images are representative of three independent experiments that gave similar results. Band intensity is indicated below the corresponding band and expressed as fold-change relative to CTRL. β-Actin and ATGL were used as loading and transfection control, respectively. (**C**) Cells were treated with Cq as previously described and Western blot analysis of LC3 and BNIP3 was performed. Bar graph refers to the densitometry BNIP3/β-Actin ratio. Band intensity of LC3 is indicated below the corresponding band and expressed as fold-change relative to CTRL. The images are representative of three independent experiments that gave similar results. Data are shown as fold change (n = 3; * p < 0.05; ** p < 0.01 as indicated*)* β-Actin and ATGL were used as loading and transfection control, respectively.

**Additional file 2 Fig. S2.** (**A**) Proliferation rate of HeLa cells over-expressing ATGL and BNIP3 ΔTM was assayed by Trypan blue direct cell counting procedure. Data are expressed as mean ± SD (n = 3; * p < 0.05; ** p < 0.01 as indicated*).* (**B**) Flow cytometry analyses of apoptosis induction in HeLa cells after 48 h of ATGL and BNIP3 ΔTM over-expression, by using Annexin-V and propidium iodide (PI) fluorescence staining assay. Each scatter plot shows the percentage of early apoptotic cells (Annexin-V + cells, lower right quadrant) and late apoptotic cells (PI + and Annexin V + cells, upper right quadrant).

**Additional file 1 Fig. S3*.*** Me-180 cells were transfected with ATGL plasmid for 48 h. (**A**) Western blot analysis of HIF1α, BNIP3 levels. Band intensity is indicated below the corresponding band and expressed as fold-change relative to CTRL. The images are representative of three independent experiments that gave similar results. β-Actin and ATGL were used as loading and transfection control, respectively. (**B**) Me-180 cells, transfected as previously described, were treated 24 h before the end of experiment with 5 mM NAC. Band intensity is indicated below the corresponding band and expressed as fold-change relative to CTRL. The image of Western blot analysis of HIF1α, BNIP3 levels, is representative of three independent experiments that gave similar results. β-Actin and ATGL were used as loading and transfection control, respectively.

## Data Availability

The dataset analysed during the current study are available in the Gene Expression Omnibus repository (GEO; http://www.ncbi.nlm.nih.gov/geo, accession number GSE63514).

## Abbreviations

ATGL: Adipose triglyceride lipase
HIF1α: Hypoxia-inducible factor-1α
FAs: Fatty acids
BNIP3: BCL2 and adenovirus E1B 19-kDa-interacting protein 3
OXPHOS: Oxidative phosphorylation
LDs: Lipid droplets
HSL: Hormone sensitive lipase
MAGL: Monoacylglycerol lipase
ROS: Reactive oxygen species
BrdU: Bromodeoxyuridine
EMT: Epithelial/mesenchymal transition
ACTB: β-actin
GAPDH: Glyceraldehyde 3-phosphate dehydrogenase
VEGF: Vascular endothelial growth factor
PNPLA2: Patatin Like Phospholipase Domain Containing 2
GLUT1: Glucose transporter
HK: Hexokinase
MCT1: Monocarboxylate transporter 1
MCT4: Monocarboxylate transporter 4
PDH: Pyruvate dehydrogenase
2DG: 2-deoxyglucose
Cpt1a: Carnitine Palmitoyltransferase 1a
MTR: MitoTracker Red
MTG: MitoTracker Green
TOM20: Translocase of outer mitochondrial membrane 20
TFAM: Transcription Factor A, Mitochondrial
Cq: Chloroquine
DRP1: Dynamin-related Protein 1
LC3: Microtubule-associated protein 1A/1B-light chain 3
LDH-A: Lactate dehydrogenase A
NAC: N-acetylcysteine
CHX: Cycloheximide
HRE: Hypoxia response elements
