# Supplementary material for: ROS-dependent HIF1α activation under forced lipid catabolism entails glycolysis and mitophagy as mediators of higher proliferation rate in cervical cancer cells
Source: J Exp Clin Cancer Res. 2021 Mar 11;40:94. doi: 10.1186/s13046-021-01887-w (PMC7948341; doi:10.1186/s13046-021-01887-w)
Supplement: Supplementary file 1 — Additional file 1 Fig. S1. HeLa cells were transfected with ATGL plasmid for 48 h. Cells were treated with the autophagy inhibitor chloroquine (Cq, 30 μM) for 2 h and (A) Western blot analysis of mitochondrial markers TFAM and TOM20 was performed. Bar graph refers to the densitometry TOM20/β-Actin and TFAM/β-Actin ratio. The images are representative of three independent experiments that gave similar results. Data are shown as fold change (n = 3; * p < 0.05; *** p < 0.001 as indicated). β-Actin and ATGL were used as loading and transfection control, respectively. (B) Western blot analysis of phosphorylated DRP1 (p-DRP1 S616) and DRP1 levels, images are representative of three independent experiments that gave similar results. Band intensity is indicated below the corresponding band and expressed as fold-change relative to CTRL. β-Actin and ATGL were used as loading and transfection control, respectively. (C) Cells were treated with Cq as previously described and Western blot analysis of LC3 and BNIP3 was performed. Bar graph refers to the densitometry BNIP3/β-Actin ratio. Band intensity of LC3 is indicated below the corresponding band and expressed as fold-change relative to CTRL. The images are representative of three independent experiments that gave similar results. Data are shown as fold change (n = 3; * p < 0.05; ** p < 0.01 as indicated) β-Actin and ATGL were used as loading and transfection control, respectively. [file 13046_2021_1887_MOESM1_ESM.pptx]

## Slide 1
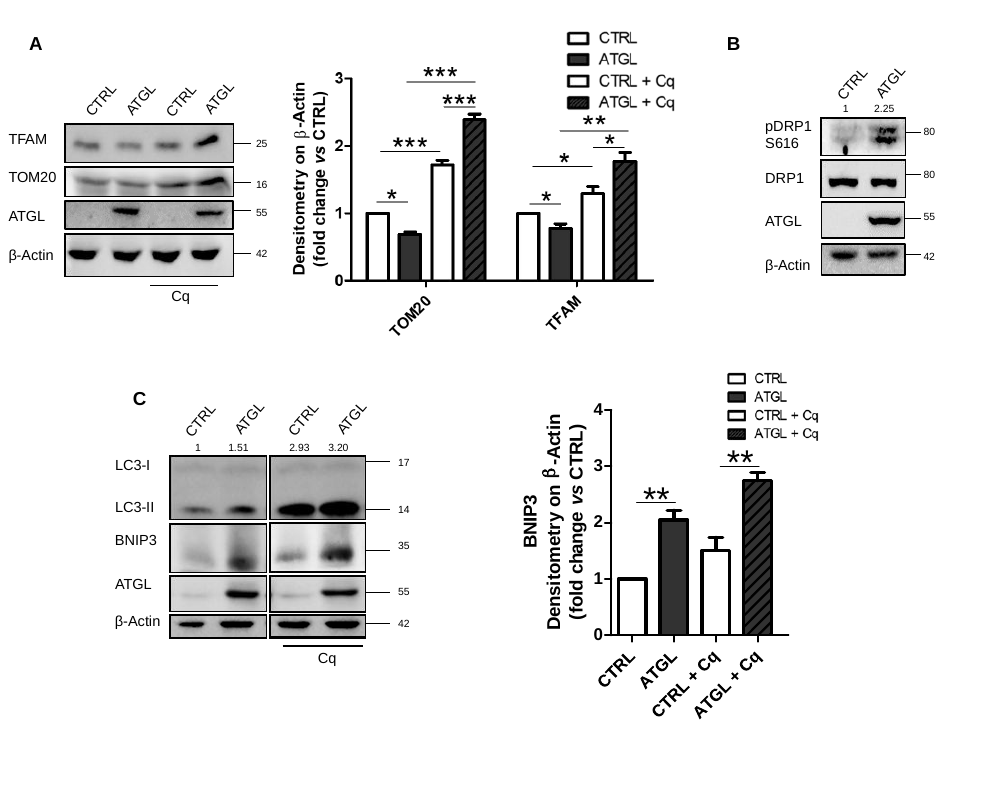

ATGL
CTRL
1
2.25
pDRP1
S616
80
80
DRP1
55
ATGL
42
β-Actin
A
B
CTRL
ATGL
CTRL
TFAM
25
TOM20
16
55
ATGL
β-Actin
42
Cq
 ATGL
 ATGL
 ATGL
CTRL
CTRL
1
1.51
2.93
3.20
LC3-I
17
LC3-II
14
BNIP3
35
ATGL
55
β-Actin
42
Cq
C
