# Supplementary figures and images for: ROS-dependent HIF1α activation under forced lipid catabolism entails glycolysis and mitophagy as mediators of higher proliferation rate in cervical cancer cells
Source: J Exp Clin Cancer Res. 2021 Mar 11;40:94. doi: 10.1186/s13046-021-01887-w (PMC7948341; doi:10.1186/s13046-021-01887-w)

## Slide 1
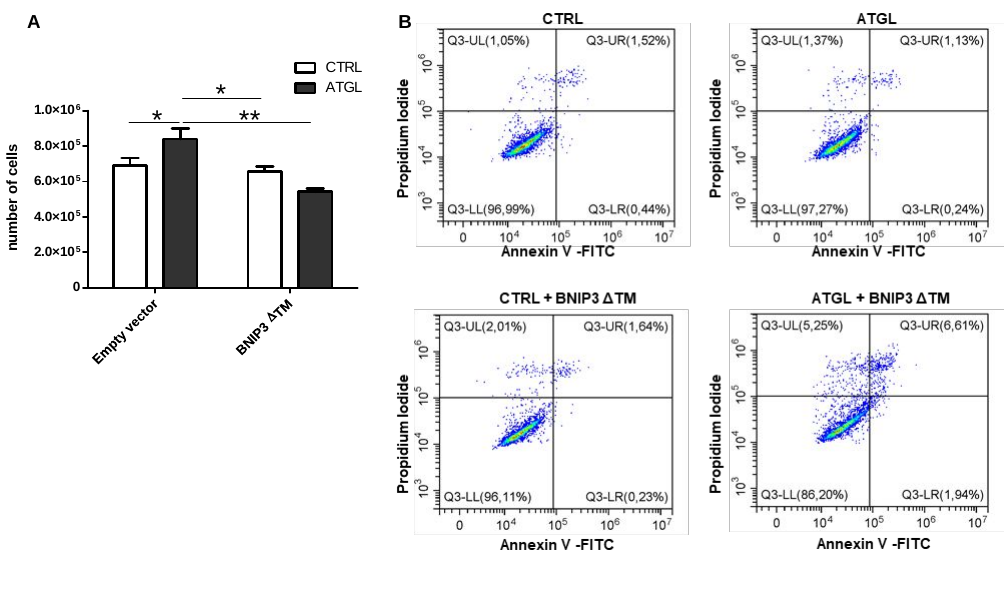

A
B

Supplement: Supplementary file 2 — Additional file 2 Fig. S2. (A) Proliferation rate of HeLa cells over-expressing ATGL and BNIP3 ΔTM was assayed by Trypan blue direct cell counting procedure. Data are expressed as mean ± SD (n = 3; * p < 0.05; ** p < 0.01 as indicated). (B) Flow cytometry analyses of apoptosis induction in HeLa cells after 48 h of ATGL and BNIP3 ΔTM over-expression, by using Annexin-V and propidium iodide (PI) fluorescence staining assay. Each scatter plot shows the percentage of early apoptotic cells (Annexin-V + cells, lower right quadrant) and late apoptotic cells (PI + and Annexin V + cells, upper right quadrant). [file 13046_2021_1887_MOESM2_ESM.pptx]

## Slide 1
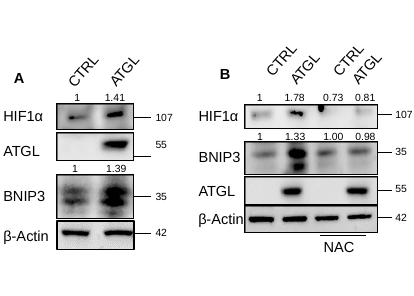

ATGL
CTRL
1
1.41
HIF1α
107
55
ATGL
1
1.39
BNIP3
35
42
β-Actin
 ATGL
 ATGL
CTRL
CTRL
1
1.78
0.73
0.81
HIF1α
107
1
1.33
1.00
0.98
35
BNIP3
ATGL
55
β-Actin
42
NAC
B
A

Supplement: Supplementary file 3 — Additional file 1 Fig. S3. Me-180 cells were transfected with ATGL plasmid for 48 h. (A) Western blot analysis of HIF1α, BNIP3 levels. Band intensity is indicated below the corresponding band and expressed as fold-change relative to CTRL. The images are representative of three independent experiments that gave similar results. β-Actin and ATGL were used as loading and transfection control, respectively. (B) Me-180 cells, transfected as previously described, were treated 24 h before the end of experiment with 5 mM NAC. Band intensity is indicated below the corresponding band and expressed as fold-change relative to CTRL. The image of Western blot analysis of HIF1α, BNIP3 levels, is representative of three independent experiments that gave similar results. β-Actin and ATGL were used as loading and transfection control, respectively. [file 13046_2021_1887_MOESM3_ESM.pptx]
